# Preparation and Evaluation of Resveratrol-Loaded Composite Nanoparticles Using a Supercritical Fluid Technology for Enhanced Oral and Skin Delivery

**DOI:** 10.3390/antiox8110554

**Published:** 2019-11-14

**Authors:** Eun-Sol Ha, Woo-Yong Sim, Seon-Kwang Lee, Ji-Su Jeong, Jeong-Soo Kim, In-hwan Baek, Du Hyung Choi, Heejun Park, Sung-Joo Hwang, Min-Soo Kim

**Affiliations:** 1 College of Pharmacy, Pusan National University, 63 Busandaehak-ro, Geumjeong-gu, Busan 46241, Korea; edel@pusan.ac.kr (E.-S.H.); popo923@pusan.ac.kr (W.-Y.S.); lsk7079@pusan.ac.kr (S.-K.L.); sui15@pusan.ac.kr (J.-S.J.); 2 Dong-A ST Co. Ltd., Giheung-gu, Yongin, Gyeonggi 446-905, Korea; ttung2nd@naver.com; 3 College of Pharmacy, Kyungsung University, 309, Suyeong-ro, Nam-gu, Busan 48434, Korea; baek@ks.ac.kr; 4 Department of Pharmaceutical Engineering, Inje University, Gyeongnam 621-749, Korea; choidh@inje.ac.kr; 5 Department of Industrial and Physical Pharmacy, College of Pharmacy, Purdue University, 575 Stadium Mall Drive, West Lafayette, IN 47907, USA; pharmacy4336@gmail.com; 6 College of Pharmacy and Yonsei Institute of Pharmaceutical Sciences, Yonsei University, 85 Songdogwahak-ro, Yeonsu-gu, Incheon 21983, Korea; sjh11@yonsei.ac.kr

**Keywords:** resveratrol, solubility, nanoparticle, correlation, supercritical fluid, bioavailability

## Abstract

We created composite nanoparticles containing hydrophilic additives using a supercritical antisolvent (SAS) process to increase the solubility and dissolution properties of *trans*-resveratrol for application in oral and skin delivery. Physicochemical properties of *trans*-resveratrol-loaded composite nanoparticles were characterized. In addition, an in vitro dissolution–permeation study, an in vivo pharmacokinetic study in rats, and an ex vivo skin permeation study in rats were performed. The mean particle size of all the composite nanoparticles produced was less than 300 nm. Compared to micronized *trans*-resveratrol, the *trans*-resveratrol/hydroxylpropylmethyl cellulose (HPMC)/poloxamer 407 (1:4:1) nanoparticles with the highest flux (0.792 μg/min/cm^2^) exhibited rapid absorption and showed significantly higher exposure 4 h after oral administration. Good correlations were observed between in vitro flux and in vivo pharmacokinetic data. The increased solubility and flux of *trans*-resveratrol generated by the HPMC/surfactant nanoparticles increased the driving force on the gastrointestinal epithelial membrane and rat skin, resulting in enhanced oral and skin delivery of *trans*-resveratrol. HPMC/surfactant nanoparticles produced by an SAS process are, thus, a promising formulation method for *trans*-resveratrol for healthcare products (owing to their enhanced absorption via oral administration) and for skin application with cosmetic products.

## 1. Introduction

*Trans*-resveratrol is abundant in various foods, such as grapes, peanuts, and berries, and is usually taken as a dietary supplement. Chemically, *trans*-resveratrol is known as 3,5,4‘-trihydroxystilbene, a non-flavonoid polyphenolic compound produced by plants in response to injury or attack by bacteria and fungi [1]. When exposed to UV light, the typically low transformation of *trans*-resveratrol to *cis*-resveratrol is accelerated [2]. *Trans*-resveratrol has been shown to have several beneficial properties, including anti-aging, anticancer, antidiabetic, anti-inflammatory, antioxidant, cardioprotective, and neuroprotective activities [3,4,5,6]. Unfortunately, due to its poor water solubility, instability, short plasma half-life, and extensive metabolism in the intestine and liver, clinical uses of *trans*-resveratrol are limited to oral administration [1,7,8,9]. *Trans*-resveratrol is a Biopharmaceutical Classification System (BCS) class II compound with an insolubility in aqueous solutions of pH 1.0 to pH 7.5 and high permeability [1]. Due to these properties, *trans*-resveratrol is quickly metabolized, therefore skin application may be an alternative to oral administration [10,11,12]. Various formulation strategies, including the use of liposomes, solid dispersions, cyclodextrin complexes, solid lipid nanoparticles, emulsions, polymeric micelles, polymeric nanoparticles, and nanocrystals, have been evaluated to attempt to overcome current limitations of *trans*-resveratrol [13,14,15,16,17,18,19,20,21]. In particular, a pharmacokinetic study of twelve healthy volunteers given an oral administration of a capsule formulation of resveratrol via solubilization with micelles consisting of polysorbate 80, polysorbate 20, and medium chain triacylglycerol yielded increases in the area under the plasma concentration versus time curve (*AUC*) and maximum plasma concentration (*C*_max_) of resveratrol of 5.0-fold and 10.6-fold, respectively, compared to volunteers ingesting a resveratrol powder [19]. In a study with rabbits, a solid dispersion of resveratrol produced an *AUC* value that was 3-fold higher compared to rabbits who ingested a resveratrol/magnesium dihydroxide solid dispersion [20]. However, in a study of resveratrol and piperine cocrystals, concentration of resveratrol in saturated solution at various conditions was decreased compared to pure resveratrol, resulting in decreased oral bioavailability [21]. It is, thus, critical to enhance the in vitro solubility and dissolution properties of resveratrol to improve its absorption by the body, and thus increase its biological performance [22,23].

We hypothesize that the rapid dissolution rate and high degree of supersaturation (solubility) of *trans*-resveratrol formulated with amorphous composite nanoparticles is directly related to increases in *trans*-resveratrol absorption. In this study, composite nanoparticles containing hydrophilic additives were produced using the supercritical antisolvent (SAS) process to increase the solubility and dissolution properties of *trans*-resveratrol for application by oral and skin delivery. Supercritical carbon dioxide (SC-CO_2_) was used as an antisolvent, and it has significant safety advantages. The airborne concentration at 25 °C, considering a high threshold limit value (TLV) of 5000 ppm, is a safe environment that a workforce may be exposed to daily without adverse effects. In addition, SC-CO_2_ is highly dense, permeable, has a high solvent power and diffusion rate, and is usually miscible with organic solvents [24]. These properties can cause a higher supersaturation during the SAS process, hence reducing the critical energy barrier for nucleation, which leads to faster nucleation, and therefore the precipitation of more and smaller particles [25]. Physicochemical characterization of composite nanoparticles was carried out using particle size and specific surface measurements, scanning electron microscopy, powder X-ray diffraction, differential scanning calorimetry, and kinetic solubility analysis. In addition, an in vitro dissolution–permeation study was performed to compare the *trans*-resveratrol flux of different composite nanoparticles prepared by an SAS process. An in vivo pharmacokinetic study in rats was also performed. We also investigated the correlation between in vitro flux data and in vivo pharmacokinetic data on *trans*-resveratrol. Finally, we performed ex vivo skin permeation studies using rats to investigate the use of *trans*-resveratrol-loaded composite nanoparticles for skin delivery.

## 2. Materials and Methods

### 2.1. Materials

*Trans*-resveratrol was supplied by Ningbo Liwah Pharmaceutical Co., Ltd. (Zhejiang, China), and micronized to a mean particle size of 2.6 μm and purity of 99.1% by using an air jet mill. Polyvinylpyrrolidone K 12/25/30/90 (PVP K12/K25/K30/K90), polyvinylpyrrolidone vinyl acetate 64 (PVP VA 64), polyvinyl caprolactampolyvinyl acetate-polyethylene glycol graft copolymer (Soluplus^®^), macrogol (15)-hydroxystearate (Kolliphor^TM^ HS 15), d-α-Tocopherol polyethylene glycol 1000 succinate (TPGS), and poly (ethylene glycol)-block-poly (propylene glycol)-block-poly(ethylene glycol) (Poloxamer 188/407) were obtained from BASF (Ludwigshafen, Germany). Hydroxylpropylmethyl cellulose (HPMC 3 cp/4.5 cp/6 cp) and low viscosity hydroxylpropyl cellulose (HPC-SSL) were provided by Shin-Etsu chemical Co., Ltd. (Tokyo, Japan), and Nippon Soda Co., Ltd. (Japan), respectively. Polyethylene glycol 6000, sodium carboxymethylcellulose (CMC), and sodium lauryl sulfate (SLS) were purchased from Sigma-Aldrich Co., Ltd. (St. Louis, MO, USA). Sucrose laurate (Ryoto^TM^ Ester L-1695, Mitsubishi-Kagaku Foods Co., Tokyo, Japan) was gifted by Namyung commercial Co., Ltd. (Seoul, Korea). Sorbitan monolaurate (Span^®^ 20), sorbitan monostearate (Span^®^ 60), sorbitan monooleate (Span^®^ 80), and polysorbate 20/60/80 (Tween^®^ 20/60/80) were purchased from Daejung Chemicals and Metals Co., Ltd. (Siheung-si, Korea). Propylene glycol dicaprolate/dicaprate (Labrafac^TM^ PG, Gattefossè, Saint-Priest, France), medium chain triglycerides (Labrafac^TM^ Lipophile WL1349, Gattefossè, Saint-Priest, France), oleoyl polyoxyl-6 glycerides (Labrafil^®^ M 1944CS, Gattefossè, Saint-Priest, France), linoleoyl polyoxyl-6 glycerides (Labrafil^®^ M 2125CS, Gattefossè, Saint-Priest, France), lauroyl polyoxyl-6 glycerides (Labrafil^®^ M 2130CS, Gattefossè, Saint-Priest, France), caprylocaproyl polyoxyl-8 glycerides (Labrasol^®^, Gattefossè, Saint-Priest, France), propylene glycol monolaurate type II (Lauroglycol^TM^ 90, Gattefossè, Saint-Priest, France), and polyglyceryl-3 dioleate (Plurol^®^ Oleique CC 497, Gattefossè, Saint-Priest, France) were kindly provided by Masung Chemicals (Seoul, Korea). All organic solvents and reagents used were either of high-performance liquid chromatography (HPLC) grade or analytical grade and were purchased from Honeywell Burdick and Jackson (Muskegon, MI, USA) or Daejung Chemicals and Metals Co., Ltd. (Siheung-si, Korea), respectively.

### 2.2. Solubility Studies of Trans-Resveratrol in Aqueous Solutions Containing Various Additives

The solubility of *trans*-resveratrol in aqueous solutions was measured to test different polymers and surfactants. First, aqueous solutions containing 1% polymer or 1% surfactant (w/v) were prepared and excess resveratrol was added to an amber vial containing 10 mL of each solution. Samples were sonicated for 1 h then incubated in a shaking water bath at 37 °C for 72 h to reach equilibrium. Next, samples were centrifuged at 12,000× *g* for 15 min and filtered through a 0.2 μm glass fiber syringe filter. Then, 1 mL of filtrate was taken into an amber volumetric flask and diluted with methanol. The concentration of resveratrol was then determined using a Shimadzu HPLC system (Shimadzu, Tokyo, Japan) consisting of an SPD-20A ultraviolet–visible (UV/VIS) detector, CBM-20A communications bus module, SIL-20AC autosampler, LC-20AT liquid chromatograph, DGU-20A 5R degassing unit, and a C18 analytical column (4.6 × 150 mm, 5 μm, Shiseido, Tokyo, Japan). HPLC analysis conditions were as follows: a 40% acetonitrile in water mobile phase, 0.8 mL/min flow rate, 30 °C column temperature, 10 μL injection volume, and UV detector wavelength of 303 nm.

### 2.3. Nanoparticle Preparation Using an SAS Process

For preparation of *trans*-resveratrol-loaded composite nanoparticles, an SAS process was applied using the equipment as previously described [26,27]. CO_2_ gas was liquefied using a cooler, raised to the required temperature using a heat exchanger, and was pumped through an ISCO^TM^ pump (Model 260D, Teledyne Technologies Inc., Thousand Oaks, CA, USA) into a high-pressure precipitation vessel. Alongside an SAS process, drug solutions were prepared by dissolving resveratrol/polymer (HPMC 6 cp)/surfactant (gelucire 44/14, TPGS, or poloxamer 407)) in a mixture of methanol and dichloromethane (1:1, w/w) in an amber vial. When the system reached a certain temperature and pressure (40 °C and 12 MPa), the drug solution was injected through the nozzle into a high-pressure vessel at a constant rate (1 g/min), along with supercritical carbon dioxide (40 g/min). The temperature of the precipitation vessel was maintained by circulating water with a temperature-controlled bath circulator. After injection of the drug solution was complete, additional carbon dioxide was applied to remove residual solvent dissolved in supercritical carbon dioxide. The pressure in the precipitation vessel was then slowly reduced to atmospheric pressure using the back-pressure regulator and the precipitated particles inside the vessel were collected.

### 2.4. Trans-Resveratrol Content Analysis

Levels of *trans*-resveratrol within the composite particles were determined using HPLC analysis of sample solutions. Composite particles were dissolved in a mixture of methanol and dichloromethane (1:1, w/w). After dilution with methanol, the concentration of *trans*-resveratrol was determined using a Shimadzu Prominence HPLC system (Shimadzu, Tokyo, Japan). The encapsulation efficiency (%) was calculated by dividing the measured concentration by the theoretical concentration and then multiplying this by 100.

### 2.5. Scanning Electron Microscopy (SEM)

The morphology of the samples was examined using a scanning electron microscope (SUPRA 25 or 40, Zeiss, Oberkochen, Germany) operating at a voltage of 5 kV. Before observation, samples were fixed to aluminum stubs with double-sided adhesive carbon tape and then gold coated at a pressure of 8–10 Pa for 1 min to increase electrical conductivity of the sample.

### 2.6. Particle Size Measurements

The average particle size of the samples was determined with dynamic light scattering (ELSZ-1000, Otsuka Electronics, Tokyo, Japan). Samples were sufficiently dispersed in mineral oil and sonicated for 10 min, then size measurements performed at least four times.

### 2.7. Specific Surface Area Measurements

The specific surface area of composite nanoparticles was measured with a Micromeritics TriStar II 3020 instrument (Micromeritics, Norcross, GA, USA), using the adsorption of nitrogen at the temperature of liquid nitrogen.

### 2.8. Differential Scanning Calorimetry (DSC)

DSC analysis was performed using a thermal analyzer (DSC25, TA instruments, Inc., New Castle, DE, USA). Prior to each analysis, temperature and heat capacity calibration were performed using high purity indium and aluminum oxide sapphire, respectively, with a temperature range of 0 to 390 °C, a modulation rate of 0.6 °C/min every 40 s, and a scan speed of 5 °C/min. Next, 2–4 mg of sample was weighed and placed in a pre-weighed aluminum hermetic pan, then sealed with an aluminum cover. Closed, empty aluminum pans were used as reference samples. Analysis was carried out by heating samples from 0 to 350 °C at a heating rate of 10 °C/min under a nitrogen purge of 400 mL/min.

### 2.9. Powder X-ray Diffraction (PXRD)

Powder X-ray diffraction analysis for samples was performed from 5° to 60° using an X-ray Diffractometer (Xpert 3, Panalytical, Almelo, Netherlands) with Ni-filtered Cu-Kα radiation. Data were collected at a scanning speed of 3°/min and a step size of 0.01.

### 2.10. Kinetic Solubility Study

A sample of 100 mg *trans*-resveratrol was placed in a water-jacked beaker containing 50 mL of distilled water maintained at 37 °C with magnetic stirring at 300 rpm. At predetermined time intervals, 3 mL of sample was withdrawn from the medium and centrifuged at 12,000× *g* for 15 min, then filtered using a 0.2 μm glass fiber syringe filter to remove insoluble material. After dilution with methanol, the concentration of *trans*-resveratrol was quantified using HPLC analysis. All sample measurements were repeated six times.

### 2.11. Flux Measurements via In Vitro Dissolution and Permeation Studies

To compare *trans*-resveratrol flux between different composite nanoparticles prepared by an SAS process and to evaluate the correlation between in vitro flux data and in vivo pharmacokinetic data for *trans*-resveratrol, a flux measurement study of *trans*-resveratrol was carried out using a miniaturized dissolution–permeation apparatus (μFLUX^TM^ apparatus, Pion Inc., Billerica, MA, USA) [28]. This apparatus contains a horizontal diffusion cell composed of a donor cell, membrane, and receiver cell. The membrane (diffusion area of 1.54 cm^2^) used to separate the donor and receiver cells was prepared by impregnating support material (polyvinylidenfluoride, 0.45 μm pore size, 70% porous, 120 μm thickness) with 50 μL of GIT^TM^ lipid solution consisting of 20% phospholipid in a dodecane lipid solution (Pion Inc., Billerica, MA, USA). The donor cell was filled with 16 mL of simulated intestinal fluid (pH 6.8, with pancreatin), while the receiver cell was filled with 16 mL of acceptor sink buffer (ASB, Pion Inc., Billerica, MA, USA), consisting of a hydroxyethyl piperazine ethane sulfonicacid (HEPES)-based pH 7.4 buffer containing surfactant micelles to ensure the sink condition of *trans*-resveratrol. The temperature of the diffusion cell was maintained at 37 °C by circulating water through the heating block with a temperature bath circulator. A 32 mg sample of *trans*-resveratrol was placed in the donor cell with magnetic stirring at 150 rpm. The concentration of drug in both the donor cell and receiver cell was quantified using UV fiber optic probes (2 mm path length for the donor cell and 20 mm path length for the receiver cell) connected with Pion Rainbow spectrometers, using a wavelength of 306 nm. Data were collected every 1 min for the first 30 min, then every 5 min for the next 240 min. The calibration curve for *trans*-resveratrol levels in simulated intestinal fluid (pH 6.8, with pancreatin) in the donor cell was generated by diluting a stock solution of *trans*-resveratrol in a mixture of ethanol and simulated intestinal fluid at pH 6.8 (with pancreatin). The calibration curve for *trans*-resveratrol levels in the receiver cell was generated using serial dilutions of a stock solution of *trans*-resveratrol in acceptor sink buffer. All sample tests were repeated four times. Flux (*J*), the mass transfer through the membrane, is calculated using Equation (1):

(1) $$J=\frac{dm}{Sdt}=\frac{V}{S}\cdot \frac{dc}{dt}$$

where *dc*/*dt* is the slope of the concentration of *trans*-resveratrol vs. the time profile in the range with linear slope (except for lag time), *V* is the volume (mL) of medium in the donor cell, and *S* is the permeation area (cm^2^).

### 2.12. Pharmacokinetic Study of Oral Delivery in Rats

The animal study protocol is in compliance with institutional guidelines for the care and use of laboratory animals and was approved by the ethics committee of Kyungsung University (No. 17-004A). To investigate the oral bioavailability of *trans*-resveratrol composite nanoparticles, the in vivo pharmacokinetics of *trans*-resveratrol in male Sprague–Dawley (SD) rats were evaluated. Thirty-six male SD rats (200 ± 10 g; Hyochang Science, Daegu, Korea) were divided into six treatment groups of six rats each. The six experimental groups received either micronized *trans*-resveratrol, *trans*-resveratrol/HPMC composite nanoparticles (1:4 or 1:5), or *trans*-resveratrol/HPMC/surfactant composite nanoparticles (1:4:1) at *trans*-resveratrol doses of 20 mg/kg by oral administration. Samples were dispersed in 1 mL of water immediately prior to oral dosing. Blood samples (approximately 0.25 mL each) were collected in heparinized tubes from the jugular vein of the treated rats at 0.25, 0.5, 0.75, 1, 1.5, 2, 4, 6, 8, and 12 h after dosing. Blood samples were centrifuged at 12,000 *× g* for 10 min at 4 °C. The amount of *trans*-resveratrol in plasma was determined using HPLC, following a previously reported analytical method [29]. Data were then used to determine the maximum plasma concentration of *trans*-resveratrol (*C*_max_), and the time required to reach *C*_max_ (*T*_max_) and the area under the plasma concentration versus time curve (*AUC*_0→12 h_) were calculated using the linear trapezoidal method. 

### 2.13. Ex Vivo Skin Permeation Study of Skin Delivery

For application of *trans*-resveratrol-loaded composite nanoparticles in skin delivery, ex vivo skin permeation studies were carried out for 24 h at 32 °C with vertical static-type Franz diffusion cells, using the skin of SD rats as the diffusion membrane. Firstly, after anesthesia, rat abdominal skin was shaved using electric and hand razors, then removed surgically. Skin samples were then cleaned of adherent subcutaneous fat and immersed in cold normal saline solution (pH 7.4) for 2 h. Powdered samples of *trans*-resveratrol-loaded composite nanoparticles were placed on the skin membrane surfaces, with effective diffusion areas of 1.86 cm^2^. Skin membrane surfaces and sampling ports were later covered with parafilm and aluminum foil to minimize the influx of the external compounds and the degradation of *trans*-resveratrol from light. The loading dose of *trans*-resveratrol was 0.5 mg/cm^2^ [30] The receptor cell was filled with 11.5 mL of a 60:40 mixture of phosphate-buffered saline (pH 7.4) and ethanol and stirred with a magnetic bar at 150 rpm to ensure uniform mixing. At predetermined time intervals, 0.2 mL of receptor medium was taken from the receptor compartment and replaced by an equal volume of fresh medium (32 °C). Samples were then centrifuged for 15 min at 12,000× *g*, and a 10 μL aliquot of the supernatant was injected into an HPLC system, as described above. The amount of *trans*-resveratrol permeated per unit area of the skin was then calculated. All sample tests were repeated six times.

### 2.14. Data Analysis

Data are expressed as mean ± standard deviation (*n* = 4 or 6). To evaluate the statistical significance of differences between groups, one-way ANOVA was carried out, followed by least significant difference (LSD) and Student-Newman-Keuls (SNK) tests using SPSS 25.0 software (IBM SPSS Statistics, IBM Corporation, Armonk, NY, USA).

## 3. Results and Discussions

**Figure 1:**
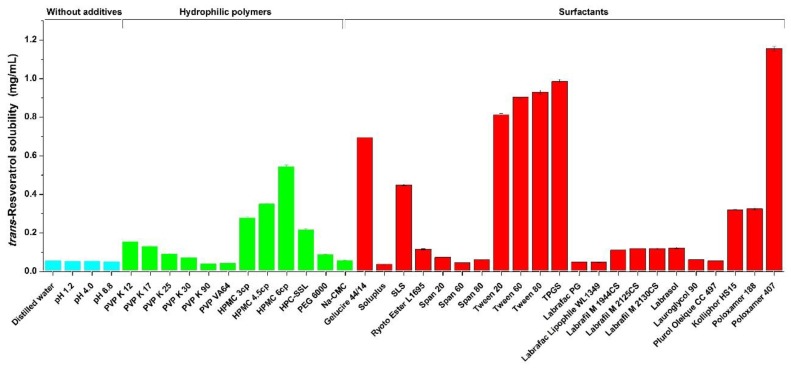
Solubility of *trans*-resveratrol in aqueous solutions containing 1% of various additives at 37 °C. Note: PVP = polyvinylpyrrolidone; HPMC = hydroxylpropylmethyl cellulose; SLS = sodium lauryl sulfate; CMC = sodium carboxymethylcellulose; HPC-SSL = hydroxylpropyl cellulose; TPGS = D-α-Tocopherol polyethylene glycol 1000 succinate; PEG = polyethylene glycol.

*Trans*-resveratrol-loaded composite nanoparticles were prepared using an SAS process with hydrophilic polymers and surfactants. For a preliminary study of various additives, the equilibrium solubility of *trans*-resveratrol in aqueous solutions containing 1% additive were determined at 37 °C and are presented in Figure 1. The solubility of *trans*-resveratrol was 55.3 μg/mL in water, 52.3 μg/mL in pH 1.2 buffer solution, 53.7 μg/mL in pH 4.0 buffer solution, and 51.1 μg/mL in pH 6.8 buffer solution. These results indicate that the solubility of *trans*-resveratrol is very poor in aqueous solution and is similar to previously reported data [31]. Among the hydrophilic polymers, *trans*-resveratrol showed the highest solubility in HPMC (6 cp). Interestingly, the solubility of *trans*-resveratrol increased with increasing viscosity of HPMC, while *trans*-resveratrol solubility showed the opposite trend in PVP. Surfactants dramatically increased *trans*-resveratrol solubility via micelle formation. The most effective surfactant tested was poloxamer 407, followed by TPGS, tween, and gelucire 44/14. The solubility of *trans*-resveratrol with poloxamer 407 was approximately 20-fold the solubility of *trans*-resveratrol alone. Based on the solubility tests, HPMC (6 cp) was selected as the polymer for nanoparticle construction and poloxamer 407, TPGS, and gelucire 44/14 were evaluated as surfactants for preparation of *trans*-resveratrol-loaded composite nanoparticles using an SAS process.

### 3.1. Preparation and Characterization of Trans-Resveratrol Composite Nanoparticles

**Figure 2:**
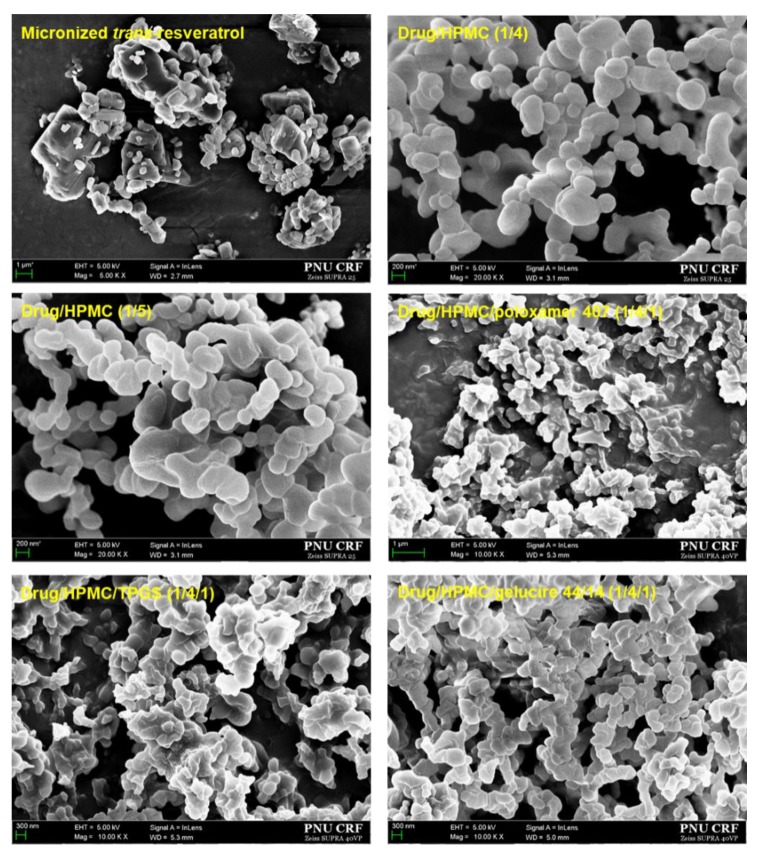
SEM images of *trans*-resveratrol composite nanoparticles.

In this study, we prepared composite nanoparticles containing 1:4 and 1:5 ratios of *trans*-resveratrol/HPMC using a SAS process and evaluated the molecular dispersion of *trans*-resveratrol within composite nanoparticles based on previously reported formulations [32]. In addition, the effects of surfactants (poloxamer 407, TPGS, and gelucire 44/14) on the physicochemical properties and in vivo performance of *trans*-resveratrol-loaded composite nanoparticles was evaluated at a 1:4:1 ratio of *trans*-resveratrol/HPMC/surfactants. In our previous work, nanoparticle agglomeration was observed in composite nanoparticles with high levels of low melting point surfactants (poloxamer 407, TPGS, and gelucire 44/14) prepared using an SAS process [33,34,35]. As shown in Figure 2 and Table 1, micronized *trans*-resveratrol morphology includes needle-shaped particles with mean particle sizes of 2.6 μm, while *trans*-resveratrol-HPMC nanoparticles are spherical particles with sizes of 180–190 nm and specific surface areas of 60–64 m^2^/g. No significant differences were observed between *trans*-resveratrol-HPMC nanoparticles composed of 1:4 and 1:5 ratios of *trans*-resveratrol/HPMC. However, surfactant addition to nanoparticles increased mean particle size and reduced specific surface areas. In particular, *trans*-resveratrol/HPMC/TPGS nanoparticles with mean particle sizes of 293.4 nm exhibited fusion and aggregations of nanoparticles with specific surface areas of 36.4 m^2^/g due to the low melting temperature of TPGS (37 °C). Nevertheless, mean particle sizes of all the composite nanoparticles produced were less than 300 nm. *Trans*-resveratrol was successfully incorporated into composite nanoparticles and the encapsulation efficiency exceeded 97% for all formulations, indicating that *trans*-resveratrol was not degraded during the SAS process (Table 1). In addition, SAS process yields were above 80% for all formulations. 

**Figure 3:**
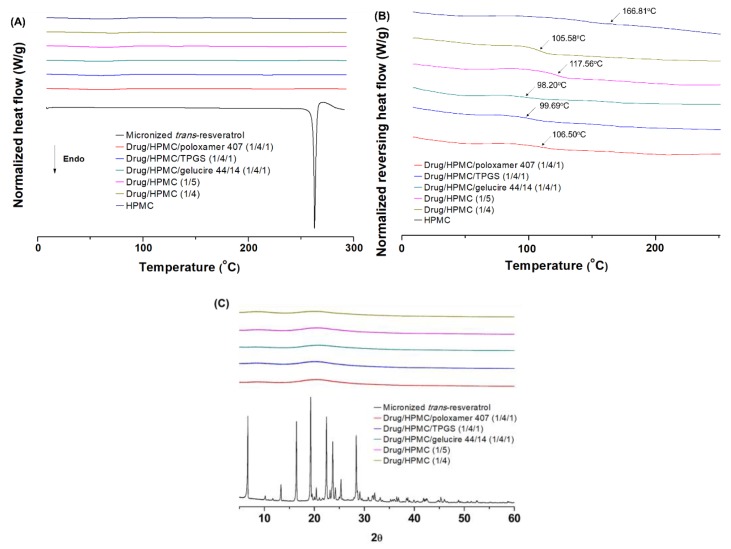
Differential scanning calorimetry thermograms of (**A**) heat flow versus temperature and (**B**) reversing heat flow versus temperature, and powder X-ray diffraction patterns (**C**) of *trans*-resveratrol composite nanoparticles.

The crystallinity and dispersion of *trans*-resveratrol in composite nanoparticles was analyzed using modulated DSC and PXRD. In DSC thermograms (Figure 3A), the melting temperature and fusion enthalpy of raw *trans*-resveratrol are 268.96 °C and 270.96 J/g, respectively, in good agreement with previously reported data [36]. The complete disappearance of a single, sharp melting endotherm for *trans*-resveratrol was observed for composite nanoparticles, indicating that *trans*-resveratrol exists in an amorphous or molecularly dispersed state within composite nanoparticles. Furthermore, we also analyzed the glass transition temperature (*T*_g_) using modulated DSC measurements. If small molecule drugs act as a plasticizer, the *T*_g_ value of the polymer should decrease with increasing drug content in polymer–drug blends. As shown in reversing heat flow versus temperature thermograms (Figure 3B), the *T*_g_ value of the composite nanoparticles decreased with increasing *trans*-resveratrol level, and all composite nanoparticles exhibited one *T*_g_ value. From these results, *trans*-resveratrol appears dispersed at the molecular level within the composite nanoparticles. In addition, samples of micronized *trans*-resveratrol and composite nanoparticles were characterized using PXRD to assess the preparation of amorphous composites of *trans*-resveratrol (Figure 3C). Micronized *trans*-resveratrol exhibited characteristic peaks at 2θ, similar to previously reported results [37] However, characteristic peak patterns for *trans*-resveratrol were not observed for all composite nanoparticle preparations, indicating that *trans*-resveratrol is molecularly dispersed within composite nanoparticles. 

**Figure 4:**
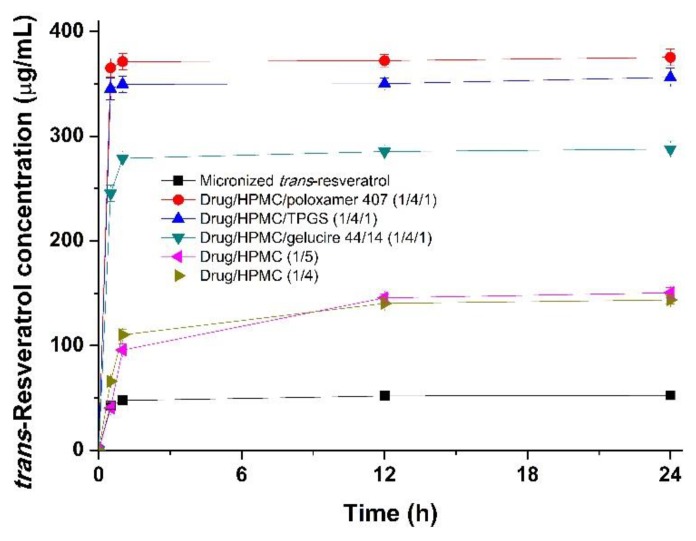
Kinetic solubility profiles of *trans*-resveratrol composite nanoparticles in distilled water at 37 °C.

The kinetic solubility of *trans*-resveratrol composite nanoparticles was determined in distilled water at 37 °C. As shown in Figure 4, the degree of solubility of *trans*-resveratrol in composite nanoparticles was dramatically increased compared to that of micronized *trans*-resveratrol. In particular, the solubility of *trans*-resveratrol/HPMC/poloxamer 407 (1:4:1) nanoparticles at 24 h was significantly higher (~7.2x) than that of micronized *trans*-resveratrol. The solubility values of *trans*-resveratrol-loaded composite nanoparticles at 24 h as ranked by the SNK test were as follows: drug/HPMC/poloxamer 407 (1:4:1) > drug/HPMC/TPGS (1:4:1) > drug/HPMC/gelucire 44/14 (1:4:1) > drug/HPMC (1:5) = drug/HPMC (1:4) > micronized *trans*-resveratrol. The maximum solubility of *trans*-resveratrol from composite nanoparticles was rapidly reached and maintained for at least 24 h through high inhibition of *trans*-resveratrol crystallization by HPMC [32,38].

### 3.2. Use of Trans-Resveratrol Composite Nanoparticles for Oral Delivery

**Figure 5:**
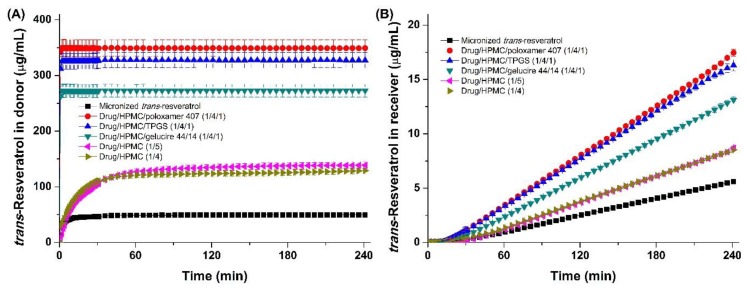
In vitro dissolution (**A**) and permeation profiles (**B**) of *trans*-resveratrol composite nanoparticles. Data are expressed as the mean ± standard deviation (*n* = 4).

To compare *trans*-resveratrol flux of different composite nanoparticles prepared by the SAS process and establish correlations between in vitro flux data and in vivo pharmacokinetic data of *trans*-resveratrol, in vitro flux measurements and in vivo pharmacokinetic experiments of *trans*-resveratrol in SD rats were carried out using the oral delivery of *trans*-resveratrol composite nanoparticles. As shown in Figure 5A, the concentration of *trans*-resveratrol from composite nanoparticles in the donor cell increased with increasing solubility of composite nanoparticles. Higher concentrations of *trans*-resveratrol in the receiver cell were observed by increasing the driving force of *trans*-resveratrol through the membrane. In addition, the concentration of *trans*-resveratrol over time in the donor cell indicated maximum dissolution of *trans*-resveratrol from the composite nanoparticles within 2 min, indicating fast dissolution from multiple HPMC/surfactant combinations. Flux (*J*) was obtained from the slope of the concentration of *trans*-resveratrol vs. time profile in the range of 30 to 240 min in Figure 5B, and is presented in Table 2. The system reached steady state within 30 min after dissolution–permeation measurements. As shown in Table 2, *trans*-resveratrol/HPMC/poloxamer 407 (1:4:1) nanoparticles exhibited the highest flux of 0.792 μg/min/cm^2^, which was 3.0-fold higher than the flux of micronized *trans*-resveratrol. Flux ranks based on the SNK test for *trans*-resveratrol-loaded composite nanoparticles were as follows: drug/HPMC/poloxamer 407 (1:4:1) > drug/HPMC/TPGS (1:4:1) > drug/HPMC/gelucire 44/14 (1:4:1) > drug/HPMC (1:5) = drug/HPMC (1:4) > micronized *trans*-resveratrol. The trend of increased flux of composite nanoparticles is similar to the trend for kinetic solubility of *trans*-resveratrol. In addition, the very fast dissolution of *trans*-resveratrol from HPMC/surfactant nanoparticles was observed in the donor cell. 

**Figure 6:**
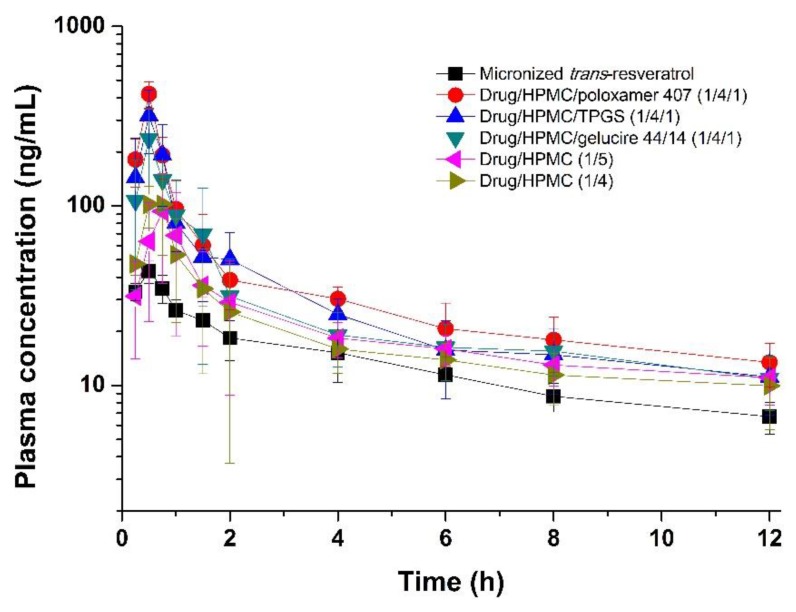
Plasma concentration versus time profiles of *trans*-resveratrol after oral administration of composite nanoparticles to Sprague–Dawley (SD) rats. Data are expressed as the mean ± standard deviation (*n* = 6).

The increased solubility and flux of *trans*-resveratrol by composite nanoparticles increases the oral bioavailability of *trans*-resveratrol [39,40]. As shown in Figure 6, HPMC/surfactant composite nanoparticles have rapid absorption rates and significantly higher exposure 4 h after oral administration compared to micronized *trans*-resveratrol. *C*_max_ ranks based on the SNK test for *trans*-resveratrol-loaded composite nanoparticles were: drug/HPMC/poloxamer 407 (1:4:1) = drug/HPMC/TPGS (1:4:1) > drug/HPMC/gelucire 44/14 (1:4:1) > drug/HPMC (1:5) = drug/HPMC (1:4) > micronized *trans*-resveratrol. The *AUC*_0–12 h_ ranked by the SNK test for *trans*-resveratrol-loaded composite nanoparticles were as follows: drug/HPMC/poloxamer 407 (1:4:1) > drug/HPMC/TPGS (1:4:1) > drug/HPMC/gelucire 44/14 (1:4:1) > drug/HPMC (1:5) = drug/HPMC (1:4) > micronized *trans*-resveratrol. Composition ranks for flux data, thus, agreed with ranks for *AUC*_0–12 h_. Greater increases in *C*_max_ and *AUC*_0→12 h_ were observed for composite nanoparticles compared to micronized *trans*-resveratrol. In particular, *C*_max_ and *AUC*_0→12 h_ of *trans*-resveratrol/HPMC/poloxamer 407 (1:4:1) nanoparticles were 9.7-fold and 3.0-fold higher, respectively, than those of micronized *trans*-resveratrol, which may be due to the rapid metabolism of *trans*-resveratrol [41].

**Figure 7:**
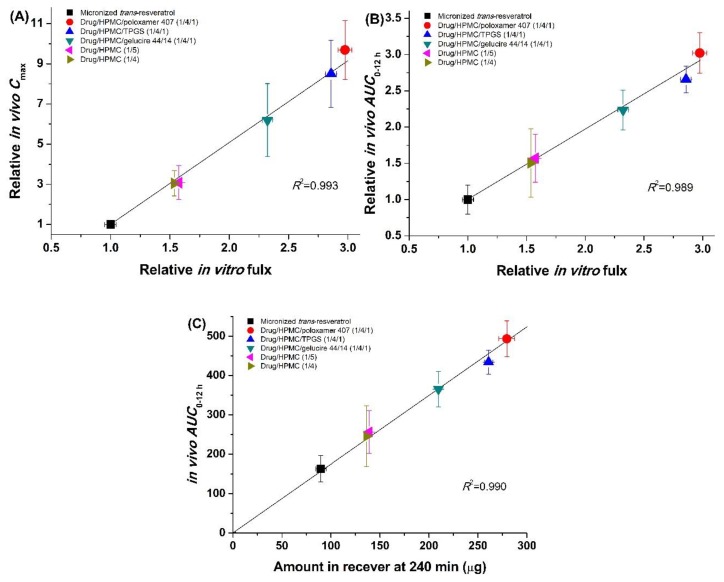
Correlations between in vitro flux data and in vivo pharmacokinetic data of *trans*-resveratrol: (**A**) in vitro flux vs. in vivo *C*_max_ of composite nanoparticles relative to micronized *trans*-resveratrol; (**B**) in vitro flux vs. in vivo *AUC*_0–12 h_ of composite nanoparticles relative to micronized *trans*-resveratrol; (**C**) total absorbed *trans*-resveratrol at 240 min in flux measurements vs. in vivo *AUC*_0→12 h_.

To further investigate correlations between in vitro flux data and in vivo pharmacokinetic data for *trans*-resveratrol, linear regression analysis was used. A good correlation was observed between relative in vitro flux, relative in vivo *C*_max_, and in vivo *AUC*_0→12 h_ of composite nanoparticles and micronized *trans*-resveratrol (*R*^2^ > 0.989). In fact, in vitro flux data reasonably represent in vivo pharmacokinetic data of *trans*-resveratrol, as previously reported for other poorly water-soluble compounds [33,34,35]. *Trans*-resveratrol/HPMC/poloxamer 407 (1:4:1) nanoparticles show 3.0-fold higher flux enhancement and 9.7-fold higher *C*_max_ compared to micronized *trans*-resveratrol. However, the3.0-fold in vivo increase in *AUC*_0→12 h_ for *trans*-resveratrol/HPMC/poloxamer 407 (1:4:1) nanoparticles relative to micronized *trans*-resveratrol more closely follows the in vitro flux enhancement. Similar to a previously reported method [28], we performed regression analysis between the total amount of *trans*-resveratrol absorbed at 240 min in flux measurements and in in vivo *AUC*_0–12 h_. At 240 min, the amounts of permeated *trans*-resveratrol in the receiver cell were 89.6 ± 2.0 μg for micronized *trans*-resveratrol, 136.4 ± 2.2 μg for drug/HPMC (1:4), 139.1 ± 2.9 μg for drug/HPMC (1:5), 209.7 ± 4.2 μg for drug/HPMC/gelucire 44/14 (1:4:1), 260.9 ± 8.2 μg for drug/HPMC/TPGS (1:4:1), and 279.5 ± 5.3 μg for drug/HPMC/poloxamer 407 (1:4:1), with the same ranks observed for in vivo *AUC*_0→12 h_. As shown in Figure 7C, there is good positive linear correlation between the total amount of *trans*-resveratrol absorbed at 240 min in flux measurements and in vivo *AUC*_0→12 h_ data obtained from plasma concentration–time profiles in rats (*R^2^* > 0.990). In fact, in vitro flux data can predict the in vivo pharmacokinetic data for *trans*-resveratrol. In addition, experimental conditions for flux measurements can be modified to establish proportional linear relationships between in vitro and in vivo data. 

Generally, the oral absorption of poorly water-soluble compounds can be accounted for by including the solubilization and dissolution processes as rate-limiting steps [42]. In this study, we demonstrated that a highly supersaturated solution generated using HPMC/surfactant nanoparticles is able to diffuse *trans*-resveratrol through membranes and enhance the flux of *trans*-resveratrol to receiver cells in in vitro flux measurement studies. Consequently, the enhanced flux of *trans*-resveratrol induces higher driving forces in the gastrointestinal epithelial membrane, resulting in enhanced oral delivery of *trans*-resveratrol in in vivo pharmacokinetic studies of rats [43,44]. Taken together, our results suggest that *trans*-resveratrol-loaded composite nanoparticles prepared using an SAS process are useful for orally delivering *trans*-resveratrol in a manner that allows fast absorption in the initial phase, resulting in higher overall exposure.

### 3.3. Utilization of Trans-Resveratrol Composite Nanoparticles for Skin Delivery

**Figure 8:**
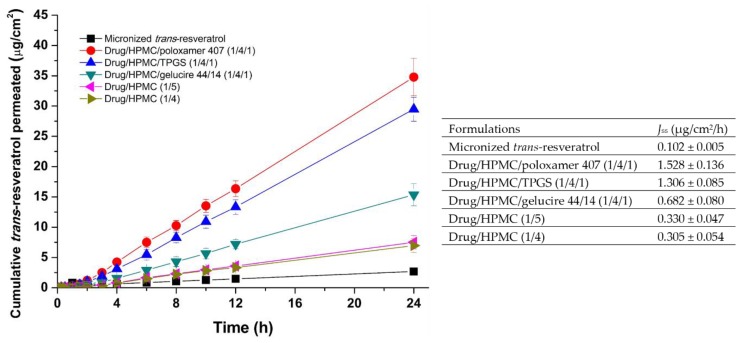
Cumulative ex vivo skin permeation profiles and flux (*J*_ss_) data for *trans*-resveratrol composite nanoparticles. Data are presented as means ± standard deviation (*n* = 6).

To investigate the application of *trans*-resveratrol-loaded composite nanoparticles for skin delivery, we performed ex vivo permeation studies using skin from rats. The cumulative amount of permeated *trans*-resveratrol (*Q*) per unit area of skin (μg/cm^2^) was determined using HPLC analysis. To calculate flux, defined as the rate of diffusion of a substance through a permeable membrane, *Q* versus time profiles were generated (Figure 8). The steady state flux (*J*_ss_) of *trans*-resveratrol was calculated based on the slope of the linear portion of the *Q* versus time profiles. The permeation of *trans*-resveratrol-loaded composite nanoparticles was higher compared to permeation of micronized *trans*-resveratrol (Figure 8). In particular, the steady state flux (*J*_ss_) of *trans*-resveratrol/HPMC/poloxamer 407 (1:4:1) nanoparticles was significantly higher (~14.9-fold) than that of micronized *trans*-resveratrol. The steady state flux (*J*_ss_) ranks for *trans*-resveratrol-loaded composite nanoparticles based on the SNK test are: drug/HPMC/poloxamer 407 (1:4:1) > drug/HPMC/TPGS (1:4:1) > drug/HPMC/gelucire 44/14 (1:4:1) > drug/HPMC (1:5) = drug/HPMC (1:4) > micronized *trans*-resveratrol. Skin penetration of *trans*-resveratrol has been confirmed in multiple studies [7,10,11]. The enhancement of solubility and dissolution rates of *trans*-resveratrol by composite nanoparticles produced using the SAS process has been shown to enhance *trans*-resveratrol penetration. Interestingly, the steady state flux (*J*_ss_) of *trans*-resveratrol from composite nanoparticles containing poloxamer 407 and TPGS was much higher than that of composite nanoparticles containing gelucire 44/14, with approximately 2.2- and 1.9-fold increases, respectively, compared to micronized *trans*-resveratrol. This increase in skin permeation is likely a result of the penetration-enhancing properties of surfactants, such as poloxamer 407 and TPGS [45,46]. Surfactants can potentially solubilize stratum corneum lipids, and thus enhance penetration [47,48]. Surfactants have well-known effects on permeability characteristics of several biological membranes, including membranes in the skin, and thus can enhance the penetration of skin by other compounds present in formulations [49]. In addition, the enhanced permeation may be due to the permeation of solute-containing nanoparticles through shunt routes, such as hair follicles [50]. However, further mechanistic studies are needed of *trans*-resveratrol-loaded composite nanoparticles.

## 4. Conclusions

In our study, we designed HPMC/surfactants nanoparticles using the supercritical antisolvent (SAS) process in order to increase solubility and dissolution properties of *trans*-resveratrol for effective oral and skin delivery. Spherical composite nanoparticles with a mean size smaller than 300 nm were successfully produced using an SAS process. Among the formulations tested, *trans*-resveratrol/HPMC/poloxamer 407 (1:4:1) nanoparticles show the highest flux of 0.792 μg/min/cm^−2^, exhibit rapid absorption, and have significantly higher exposure 4 h after oral administration than micronized *trans*-resveratrol. Good correlations between in vitro flux and in vivo pharmacokinetic data were also observed. The increased solubility and flux of *trans*-resveratrol generated using HPMC/surfactant nanoparticles increased driving forces on the gastrointestinal epithelial membrane and rat skin, resulting in enhanced oral and skin delivery of *trans*-resveratrol. HPMC/surfactant nanoparticles produced by the SAS process are, therefore, promising formulations enhancing *trans*-resveratrol absorption via oral administration for use in healthcare products, as well for skin application of cosmetic products.

## Figures and Tables

**Table 1:** Composition, encapsulation efficiency, mean particle size, and specific surface area of *trans*-resveratrol composite nanoparticles.

Formulation	Encapsulation Efficiency (%)	Mean Particle Size (nm)	Specific Surface Area (m^2^/g)
Micronized *trans*-resveratrol	-	2631.4 ± 203.1	2.98 ± 0.3
Drug/HPMC/poloxamer 407	98.9 ± 0.6	258.5 ± 19.5	40.2 ± 1.6
Drug/HPMC/TPGS	99.1 ± 0.8	293.4 ± 16.9	36.4 ± 1.1
Drug/HPMC/gelucire 44/14	97.2 ± 1.2	291.7 ± 15.2	33.1 ± 1.3
Drug/HPMC (1:5)	98.2 ± 0.9	188.2 ± 6.9	60.4 ± 2.3
Drug/HPMC (1:4)	99.9 ± 0.3	181.5 ± 8.5	63.2 ± 1.9

Data are expressed as the mean ± standard deviation (*n* = 4).

**Table 2:** In vitro flux data and in vivo pharmacokinetic data for *trans*-resveratrol composite nanoparticles.

Formulation	In Vitro Flux (μg/cm^2^/min)	In Vivo Pharmacokinetic Data		
		AUC_0→12 h_ (ng·h/mL)	C_max_ (ng/mL)	T_max_ (h)
Micronized *trans*-resveratrol	0.266 ± 0.006	163.4 ± 33.1	43.4 ± 6.3	1.0 ± 0.5
Drug/HPMC/poloxamer 407	0.792 ± 0.013 ^a,b,c,d^	493.6 ± 45.4 ^a,b,c,d^	420.3 ± 63.4 ^a,b,c^	0.5 ± 0.1
Drug/HPMC/TPGS	0.761 ± 0.016 ^a,b,c^	434.3 ± 30.1 ^a,b,c^	368.7 ± 72.1 ^a,b^	0.5 ± 0.2
Drug/HPMC/gelucire 44/14	0.618 ± 0.012 ^a,b^	365.4 ± 45.0 ^a,b^	268.7 ± 78.9 ^a^	0.7 ± 0.4
Drug/HPMC (1:5)	0.419 ± 0.012 ^a^	256.6 ± 53.8 ^a^	133.5 ± 36.7	0.9 ± 0.3
Drug/HPMC (1:4)	0.409 ± 0.004 ^a^	245.9 ± 76.9 ^a^	132.2 ± 27.3	0.7 ± 0.2

Note: ^a^ *p* < 0.05 vs. micronized trans-resveratrol; ^b^ *p* < 0.05 vs. drug/HPMC (1:5); ^c^ *p* < 0.05 vs. drug/HPMC/Gelucire 44/14 (1:4:1); ^d^ *p* < 0.05 vs. drug/HPMC/TPGS (1:4:1). Data are expressed as the mean ± standard deviation (*n* = 4 or 6). *AUC*_0→12 h_, the area under the plasma concentration versus time curve; *Cmax*, the maximum plasma concentration of trans-resveratrol; *T*_max_, the time required to reach *C*_max_.
